# A human *in vitro *model system for investigating genome-wide host responses to SARS coronavirus infection

**DOI:** 10.1186/1471-2334-4-34

**Published:** 2004-09-09

**Authors:** Lisa FP Ng, Martin L Hibberd, Eng-Eong Ooi, Kin-Fai Tang, Soek-Ying Neo, Jenny Tan, Karuturi R Krishna Murthy, Vinsensius B Vega, Jer-Ming Chia, Edison T Liu, Ee-Chee Ren

**Affiliations:** 1 Genome Institute of Singapore, 60 Biopolis Street, Genome, #02-01, Singapore 138672; 2 Department of Microbiology, Faculty of Medicine, National University of Singapore, Block MD4, 5 Science Drive 2, Singapore 117597; 3 Enviromental Health Institute, National Environment Agency, 41 Science Park Road, #03-24/28, The Gemini, Singapore Science Park II, Singapore 117610

## Abstract

**Background:**

The molecular basis of severe acute respiratory syndrome (SARS) coronavirus (CoV) induced pathology is still largely unclear. Many SARS patients suffer respiratory distress brought on by interstitial infiltration and frequently show peripheral blood lymphopenia and occasional leucopenia. One possible cause of this could be interstitial inflammation, following a localized host response. In this study, we therefore examine the immune response of SARS-CoV in human peripheral blood mononuclear cells (PBMCs) over the first 24 hours.

**Methods:**

PBMCs from normal healthy donors were inoculated *in vitro *with SARS-CoV and the viral replication kinetics was studied by real-time quantitative assays. SARS-CoV specific gene expression changes were examined by high-density oligonucleotide array analysis.

**Results:**

We observed that SARS-CoV was capable of infecting and replicating in PBMCs and the kinetics of viral replication was variable among the donors. SARS-CoV antibody binding assays indicated that SARS specific antibodies inhibited SARS-CoV viral replication. Array data showed monocyte-macrophage cell activation, coagulation pathway upregulation and cytokine production together with lung trafficking chemokines such as IL8 and IL17, possibly activated through the TLR9 signaling pathway; that mimicked clinical features of the disease.

**Conclusions:**

The identification of human blood mononuclear cells as a direct target of SARS-CoV in the model system described here provides a new insight into disease pathology and a tool for investigating the host response and mechanisms of pathogenesis.

## Background

The causative agent for SARS has been identified as a novel coronavirus [1-3] with genome sequence revealing no strong homology to existing known coronaviruses [4-6]. Coronaviruses belong to the family of enveloped viruses called *Coronaviridae*, and have the largest known single-stranded viral RNA genomes (27 to 32 kb). Coronaviruses, have both "early" and "late" phases of gene expression. Regulatory proteins are synthesized as "early" non-structural proteins, while the structural proteins are synthesized as "late" proteins. "Late" structural proteins are usually required in greater amounts thus, there is a necessity to regulate the expression of the viral genes quantitatively. After the viral entry via endocytosis or through specific receptors, the 5'-end of the viral genome is translated directly giving rise to twenty-three viral proteins, including the RNA dependent RNA polymerase (RdRp), and other functional products involved in transcription, replication, viral assembly and cell death. Coronaviruses can be classified into species and three major antigenic groups based on, serology, natural hosts, monoclonal antibody recognition and nucleotide sequencing [7]. Most coronaviruses have restricted host ranges as they infect only one host species or, at most, a few related species, they are an important group of animal pathogens. Group one (I) includes human coronavirus 229E (HCoV), porcine transmissible gastro-enteritis virus (TGEV) and feline enteric coronavirus (FECoV). Group two (II) includes bovine coronavirus (BCoV), murine hepatitis virus (MHV), and HCoV-OC43; and Group three (III) includes avian infectious bronchitis virus (IBV) [7]. Some coronaviruses like HCoV have restricted tissue tropism, including macrophages [8], although most strains that infect humans cause only mild respiratory infections.

However, SARS has rapidly caused a world-wide problem. The earliest known cases of SARS was reported in Guandong Province, China in November 2002, becoming more widespread by March 2003, when it was introduced to Canada, Singapore, Taiwan and Vietnam via Hong Kong. The largest number of infected patients has been in China with a worldwide incidence totalling more than 8,400 by July 2003. Infection by the virus induces high morbidity and mortality, the latter being estimated at 15% by the World Health Organisation. SARS is characterized by high fever, non-productive cough or dyspnea and in many cases may progress to generalized, interstitial infiltrates in the lung, thus needing intubation and mechanical ventilation [2]. The characteristic compression of alveolar sacs seen in atypical pneumonia is largely due to fluid build up outside the alveoli. One possible cause of this could be interstitial inflammation, following a localised host response. To date, the details of the host response to SARS-CoV infection is still largely unknown and consequently the most appropriate treatment regime remains to be established. Typically a pro-inflammatory cytokine profile (up regulated TNFα, Il1, IL6 and IFNs) is seen in viral infections such as influenza [9], together with perhaps limited amounts of IL8 and other chemokines [10] that may depend on which cell type is infected [11]. In experimental systems the immediate innate immune response has been shown to be directed by the monocyte-macrophage-dendritic lineage to a range of different organisms [12,13] and consists of a core set of pathways common to all, together with pathogen specific pathways. This data points to critical time points in the response, with the first 12 hours representing primary events while longer periods the consequence of this activity and a secondary (perhaps larger) cascade of responses.

We postulated that the pulmonary damage in SARS may not be a direct effect of the virus on the alveoli, but represents a secondary effect of cytokines or other factors proximal to but not from the lung tissue mediated by the host either as the primary or secondary response [2,14]. In this study, we have addressed this question by developing a human *in vitro *model system that will in the future allow detailed investigations of the host response to be made.

## Methods

### Cell culture and virus infection

PBMCs were obtained by Ficoll-Hypaque separation of whole blood. 2 × 10^5 ^PBMCs were seeded into each well of a 24-well culture plate, 0.5 ml of complete RPMI-1640 (Life Technologies-Invitrogen, USA) added to each and cultured overnight at 37°C (5% CO_2_). A seed stock of SARS-CoV (strain SIN 2774) passaged in Vero E6 cells was used for infection. Vero E6 culture supernatants were added to each well in 50 μl volume at a concentration of 0.1 or 0.01 MOI (based on plaque forming units) and a control plate (media only). Each culture was set up in duplicate. After 4 hours of incubation, one set of the duplicate wells of the control plate and a 4 hours incubation plate were harvested while the rest received 0.5 ml media top-up and incubated for a further 2, 4, 6 and 8 days.

### Cell harvest and RNA isolation

Harvesting was performed by gently flushing the wells with a Pasteur pipette to removed non-adherent cells, followed by a rinse of 1 ml RPMI. The rinsed fraction was pooled with the first harvest aliquot and spun at 1500 rpm. The cell pellet was washed twice with 2 ml RPMI to remove virus in the supernatant. 1.5 ml Trizol (Invitrogen, USA) was then added to the adherent cell fraction as well as the non-adherent cell fraction to lyse cells and stabilize the RNA. Extraction of total RNA was then performed following manufacturer's protocol and the resultant RNA dissolved in 40 μl water.

### Real-time quantitative polymerase chain reaction (PCR)

The amount of SARS-CoV in each cell fraction was measured by real-time quantitative PCR assay. 2 μl of RNA was reverse transcribed and amplified in 20 μl using 0.9 μM each of forward (5'-GGTTGGGAT TATCCAAAATGTGA-3') and reverse (5'-AGAACAAGAGAGGCCATTATCCTAAG-3') primers, and 0.25 μM of TaqMan^® ^MGB probe: 5'-(6-FAM)AGAGCCATGCCTAACAT(NFQ)-3') in a one step PCR using master mix from Applied Biosystems (USA) according to manufacturers' recommendations. Reactions were performed using an ABI PRISM 7900 sequence detection system (48°C for 30 min, followed by 95°C for 10 min and 40 cycles of 95°C for 15 sec and 60°C for 1 min) and quantitation achieved using standard curves generated from *in vitro *transcribed RNA.

### High density oligonucleotide array hybridization

At each time point (4 hours, 8 hours, 12 hours, 24 hours), 5 × 10^7 ^cells of mock-infected and infected cells were harvested and lysed using Trizol (Invitrogen, USA). Total RNA was isolated according to the manufacturer's recommendation. Quality of the total RNA was judged from the ratio between 28S and 18S RNA after agarose gel electrophoresis. 20 μg of total RNA was labeled with Cy-3 or Cy-5 using the Superscript II reverse-transcription kit (Invitrogen, USA) and hybridization was carried out overnight (16 hours) at 42°C on high-density oligonucleotide arrays (~19,200 gene features, Compugen) using universal human reference (Stratagene, USA) as a reference. Hybridized arrays were scanned at 5 μm resolution on a GenePix 4000A scanner (Axon Instruments) with variable photo-multiplier tube voltage to obtain maximal signal intensities, and the resulting images were analyzed via GenePix Pro v4.0 (Axon Instruments) as described in the manual.

### Microarray data analysis

Raw data were analyzed on GenePix analysis software version 4.0 (Axon Instruments) and uploaded to a relational database. The logarithmic expression ratio for a spot on each array was normalized by subtracting the median logarithmic ratio for the same array. Data were filtered to exclude spots with a size of less than 25 μm and any poor quality or missing spots. Since the correlation of the overall data from reciprocal labeling was good, values obtained from reciprocal labeling experiments were averaged. In addition, the data were distilled to the set of gene features that were present at all 4 time points in both the viral infected samples and the negative controls. The results were represented as the logarithmic ratio of gene expression between the viral infected samples and their corresponding negative controls at the various time points. Application of these filters resulted in the inclusion of ~12,900 of the total ~19,200 gene features in subsequent analyses.

To discover patterns of gene expression, the values associated with each gene feature *f *were translated so that their means were zero. Similar genes, whose translated gene-features exhibited same induction-repression pattern, were grouped together. Genes *g*_*i*_, *g*_*j *_were said to be similar if they satisfied the following condition:


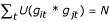
, where *U*(*x*) = 1 if *x *> 0, *U *(*x*) = 0 otherwise,

where *g*_*it *_and *g*_*jt *_denote the translated values of gene features *g*_*i*_, *g*_*j *_at time *t *respectively; and *N *is the number of time points for which the expression of a gene was observed. Similar genes, based on the above criteria, are grouped together. Within each group, the genes were ordered in the descending order of their expression range (defined as the difference between the maximum and minimum ratios of gene expression). This algorithm is a special case of the *Friendly Neighbor *algorithm currently under development. The final plots were generated using the original expression ratios while preserving the clustering and ordering discovered by the above algorithm. To determine whether a gene observed to be responsive could appear merely by chance, 100,000 expression profiles were generated by randomly sampling the expression ratios from the entire dataset with replacement. The *P *value of a gene is the fraction of the random profiles whose logarithmic expression range is as good as, or better than that of the selected gene.

## Results and Discussion

### Kinetics of SARS-CoV replication

**Figure 1:**
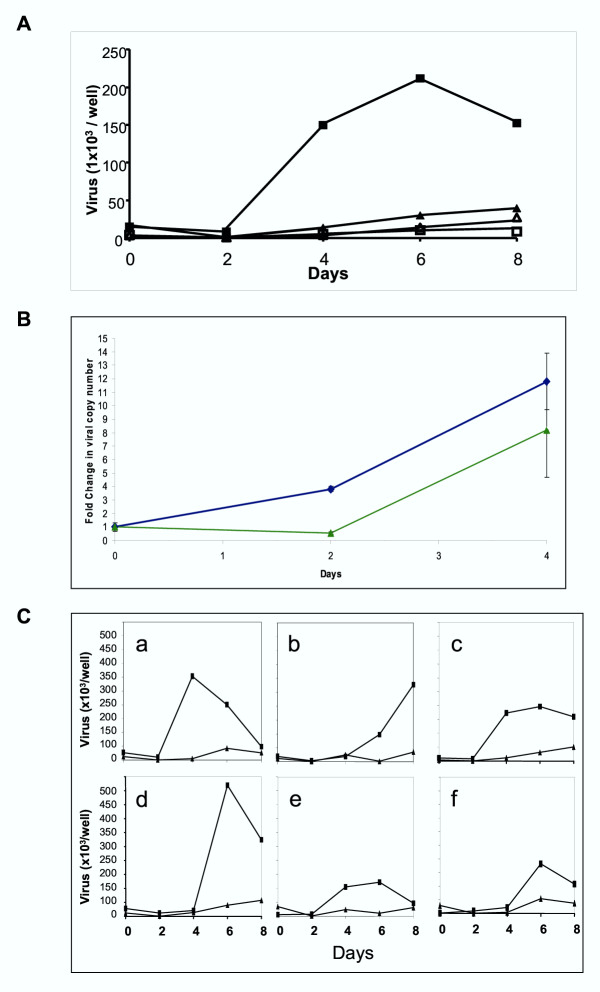
Kinetics of SARS-CoV replication. A. Viral copy number per well during culture. Two MOI infecting doses were used, 0.1 (▲,■) and 0.01 (□,△) with 2 × 10^5 ^PBMCs/well. Harvested PBMCs were separated into two fractions, adherent cells (mostly macrophages), (■,△) and suspended cells (mostly lymphocytes), (▲,□). Points represent the mean of six subjects, each done in duplicate. Increasing viral copy number is clearly seen in adherent cells using an MOI of 0.1. There was little increase in viral load in non-adherent cells infected with SARS- CoV. B. Replication of SARS-CoV in the THP-1 cell line. Using an MOI of 0.1, fold increase from day 0 in SARS-CoV infected THP-1 cells (◆) was similar to the PBMCs adherent fraction (▲). Infections were done in duplicate and assayed in duplicate. Error bars represent SEM. C. Variation in the PBMC response to SARS-CoV infection. Using an MOI of 0.1 adherent cells (■) but not suspended cells (▲) showed differing lag phase length and viral replication in 6 subjects (a to f). Points show means of two replicates.

We obtained PBMCs from 6 healthy volunteers by Ficoll-Hypaque separation of whole blood. Of the 6 donor PBMCs tested, all were able to support SARS-CoV replication when infected with multiplicity of infection (MOI) of 0.1. The first sampling, taken from cells infected for 4 hours, showed an average copy number of 32 × 10^3 ^(Fig. 1A) and represents the initial inoculum level. Over the course of the next 8 days, there was a steady rise in viral load, reaching as high as 480 × 10^3 ^copies per well in one donor, which could only be explained by active replication of the SARS-CoV intracellularly. This work is supported by recent *in vivo *evidence suggesting that SARS-CoV may have infected and replicated within PBMCs of SARS patients [15] and cells from humans and animals [16]. An indication of the PBMCs lineage involvement was provided by repeating the experiment using the monocyte-macrophage cell line THP-1 [17], in which viral replication was similar to the primary cell culture over the first 4 days (Fig. 1B). In the primary cultures, the non-adherent cell fraction which comprises mainly lymphocytes and granulocytes showed dramatically less viral replication in our assay as did all cells infected at MOI of 0.01 (Fig. 1A).

The kinetics of viral replication was variable among the 6 donors (Fig. 1C). There was a lag phase of 2 days in the case of donors a, c and e; and 4 days for donors b, d and f before any significant increase could be detected. The viral replication generally peaked at either day 4 or day 6. The exception was donor b, in which the virus seemed to replicate at a much slower pace compared to the other 5 donor samples. Equally interesting was the different levels of virus attained. Donor d seems to stand out from the rest, reaching a peak of 480 × 10^3 ^copies per well which is 4 times more than that attained by donor e, with 120 × 10^3 ^copies per well. Such variation strongly suggests that there is an underlying host-pathogen interaction influencing the kinetics of SARS-CoV replication efficiency. These *in vitro *observations may reflect the wide range of patient outcomes after SARS-CoV infection [18].

Antibody blocking experiments were also performed in which SARS-CoV was pre-incubated with convalescent patient sera for 30 minutes before introduction to the PBMCs and after a 4 day incubation period, the adherent cell fraction was harvested and assayed for SARS-CoV viral titer. Results clearly showed that even at high dilution, convalescent sera inhibited SARS viral replication (data not shown), presumably by blocking viral entry. This supports other reports indicating that SARS-CoV is not endocytosed through antibody mediated mechanisms and confirms a protective role for antibodies elicited either by the infection or through immunization [19,20].

### SARS-CoV specific gene expression changes

**Figure 2:**
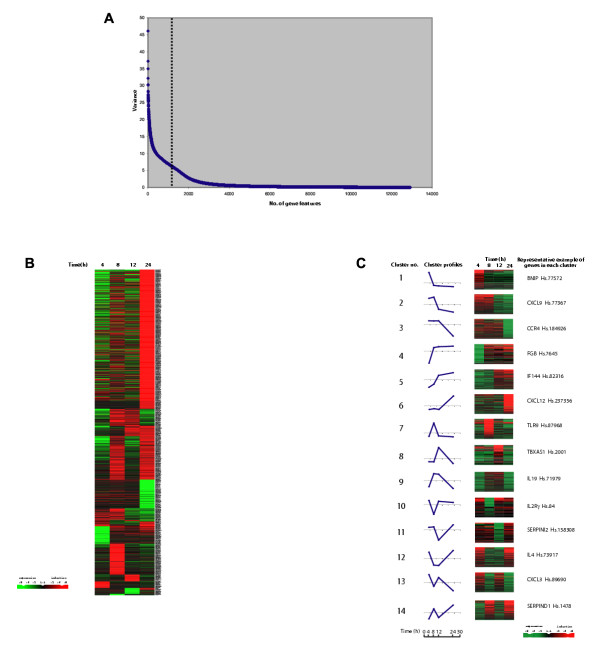
SARS specific gene expression changes. A. Plot showing the variance of expression ratio for each of the gene features across all the time points. Dotted line indicates the ~1200 most variable gene features. B. Expression ratios of ~1200 gene features as grouped by the algorithm as discussed in Methods. Rows represent individual genes, columns represent individual time points. Each cell in the matrix represents the mean expression level from 3 subjects for a gene feature at a particular time point (non-infected PBMCs responses have been subtracted from infected responses). The red and green color bars reflect high and low expression levels respectively, while black indicates equivalent expression level. The magnitude of the log-transformed ratio is reflected by the degree of color saturation. C. Levels of PBMCs mRNA expression of 1087 immune-related genes at 4 hours, 8 hours, 12 hours, and 24 hours in response to SARS-CoV infection were grouped and ordered using the algorithm described in Methods. Rows represent individual genes, columns represent individual time points. Each cell in the matrix represents the mean expression level from 3 subjects for a gene feature at a particular time point (non-infected PBMCs responses have been subtracted from infected responses). The red and green color bars reflect high and low expression levels respectively, while black indicates equivalent expression level. The magnitude of the log-transformed ratio is reflected by the degree of color saturation. The line graph indicates the average expression ratios for each group. The area above the axis indicates upregulation, while the area under the axis means downregulation.

To further elucidate the molecular processes of SARS-CoV infection, PBMCs from 3 healthy individuals were infected separately *in vitro *with SARS-CoV (0.1 MOI) and harvested at 4 hours, 8 hours, 12 hours and 24 hours time intervals post-infection. As controls, uninfected aliquots of the same PBMCs were also harvested at the corresponding time points. Total RNA extracted from the PBMCs of the 3 individuals were pooled, labeled and hybridized to human oligonucleotide arrays consisting of ~19,200 gene features. Reciprocal dye swap replicate hybridizations were performed to minimize technical noise. Analysis of variance in expression levels for each gene across all the time points indicated the ~1200 genes which showed the largest variability (Fig. 2A and 2B).

In order to focus the analysis, we queried the entire data set for genes related to the immune response by keyword searches on their gene ontology descriptions with the aim of describing the specific host-pathogen interaction. In common with other studies of respiratory pathogens [9-13], our data points towards two critical time points in the response, with the first 12 hours representing a primary pro-inflammatory cytokine profile while longer periods represent the consequence of this activity and a secondary cascade of responses [9-13]. We observed that within the first 12 hours of SARS-CoV infection, evidence of this monocyte-macrophage activation was seen, indicated by enhanced expression of CD14, TLR9 plus NFKβ1 and GATA signaling (Fig. 2C and Table 1). In addition, the MRC2 endocytotic receptor was upregulated as was the complement pathway (C1q, C6). Taken together, these data suggest an early activation of the innate immunity pathway. This activation was accompanied by an unusual cytokine transcriptional profile (Fig. 2C and Table 1). While IL1β (up regulated for the first 12 hours) would be expected following macrophage activation [21], TNFα, IFNγ and IL6 were noted by their surprisingly low level of expression. This is in spite of the presence of elevated IL19 which is thought to enhance their up regulation [22]. In some clinical investigation, concentrations of TNF and IL 6 measured during active disease were found to be relatively low [23,24], reflecting our findings. This paper did not report on IFN levels, however, we found them to be low (Supplementary figure [see Additional file 1]). This is of particular interest as IFNs have been shown to have significant anti-SARS-CoV effects [25]. Such effects suggest that alteration of the IFN response and perhaps other immune modulators might provide opportunity for novel treatment and management regimes for SARS patients to be developed.

A number of CC chemokines (CCL4, CCL20, CCL22, CCL25, CCL27) and their receptors (CCR4 and CCR7) were highly expressed in response to the infection (Fig. 2C and Table 1), indicating a rapid mobilization and increased trafficking, in particular of the monocyte-macrophage lineage very early on in the infection [26]. CXC chemokines (CXCL9, CXCL12) were also highly expressed suggesting significant increase in neutrophil homing as well. These are likely to be lung directed as IL8 and IL17 were also highly expressed [27-32]. Specific trafficking of these cells to the lung may account for the localized nature of the response [33].

Surprisingly, a number of blood coagulation genes were highly expressed early during our *in vitro *infection (Fig. 2C and Table 1), in particular TBXAS, which has been implicated in vasoconstriction, platelet aggregation, membrane lysis and increased permeability [34,35]; fibrin (FGB and FGG) and the coagulation pathway directly (SERPINs D1 and A3 together with Factors 10, 3 and 2). This gives a pro-coagulation profile, which mimics the clinical-pathological observations: at autopsy, many SARS patients have unusually disseminated small vessel thromboses in the lungs without evidence of disseminated intravascular coagulation [1,36]. Again, these expression profiles provide an experimental framework to explore an important aspect of SARS pathobiology and treatment.

**Figure 3:**
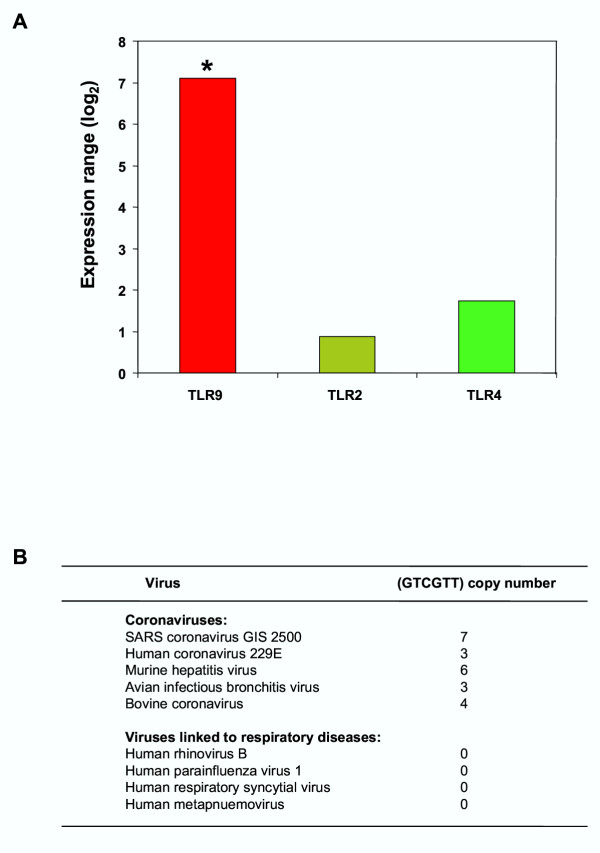
Expression of TLR9 in response to SARS-CoV infection. A. Expression range (log_2_) for TLR9, TLR2 and TLR4. The expression range for TLR9 was greater than expected (* represents a *P*-value for TLR9 of 0.016). B. Comparison of the CpG motif (GTCGTT) copy number in coronaviruses and other viruses linked to respiratory diseases. Accession numbers are as follows: SARS coronavirus SIN2500 – AY283794, Human coronavirus 229E – NC_002645, Murine hepatitis virus – NC_001846, Avian infectious bronchitis virus – NC_001451, Bovine coronavirus – NC_003045, Human rhinovirus B – NC_001490, Human parainfluenza virus 1 – NC_003461, Human respiratory syncytial virus – NC_001781, Human metapneumovirus – NC_004148.

It is interesting to note that the TLR9 was highly expressed in comparison to other TLR receptors, implying some degree of TLR specificity for the virus (Fig. 3A). TLR9 is known to respond to CpG signaling motifs (GTCGTT) [37-39] and one possibility is that the virus is activating directly through this mechanism. In support of this, we found that the SARS-CoV viral sequence contains the highest number (7 copies) of such specific signaling motifs compared to other coronaviruses and significantly more than several other viruses involved in respiratory diseases (Fig. 3B). It is conceivable that TLR9 may be aiding host recognition of the virus via the CpG groups and contributing to the initiation of the innate host inflammatory response. An alternative explanation is that TLR9 is being stimulated by mechanisms unrelated to CpG recognition.

The emerging picture from this study implicates a central role for the immune response in the pathobiology of a SARS infection. While detailed *in vivo *studies of the host response are now required, the *in vitro *model described here will allow responses to specific modulators (such as therapeutics) to be investigated. In future developments of the model, it will be interesting to compare the host response to different SARS-CoV isolates with inactivated preparations of the virus. In other diseases, *in vitro *models have revealed a number of key processes relevant to the clinical diseases [9,12,13] and it is likely that the responses identified here will prove to be equally important. Although some clinical parameters have now been used as prognostic markers [40-42], further study of the regulatory mechanisms for chemokine-cytokine production will likely improve their accuracy and perhaps allow development of new treatment protocols.

## Competing interests

None declared.

## Authors' contributions

LFPN, MLH, ETL and REC conceived the study, its design and coordination, results analysis and drafted the manuscript. EEO and KFT carried out the virus infections. SYN, KRKM, VNV and JMC were involved in the array and statistical analysis. JT carried out the real-time PCR assays.

## Pre-publication history

The pre-publication history for this paper can be accessed here:



## Supplementary Material

Additional File 1 **List of all immune related genes after SARS-CoV infection **Comprehensive list of 1087 immune related genes that were altered in PBMCs in response to SARS-CoV infection at 4 hours, 8 hours, 12 hours, and 24 hours. Genes were grouped and ordered using the algorithm described in Methods. Rows represent individual genes, columns represent individual time points. Each cell in the matrix represents the mean expression level from 3 subjects for a gene feature at a particular time point (non-infected PBMCs responses have been subtracted from infected responses). The red and green color bars reflect high and low expression levels respectively, while black indicates equivalent expression level. The magnitude of the log-transformed ratio is reflected by the degree of color saturation. The line graph indicates the average expression ratios for each group. The area above the axis indicates upregulation, while the area under the axis means downregulation. Click here for file

## Figures and Tables

**Table 1:** Representation of selected immune-related genes upregulated during the first 12 hours post-infection.

**Gene**	**Unique Gene ID.**	**Function**	**Expression range (log_2_)**	**Cluster no.**
***Immune Response Genes:***				
TGFBR1	Hs.220	Cytokine proinflammatory	10.66	4
CD36	Hs.75613	Macrophage receptor	8.61	3
TLR 9	Hs.87968	Macrophage activation	7.11	7
MST	Hs.349110	Macrophage activation	7.07	6
IL 19	Hs.71979	Cytokine, proinflammatory	6.86	9
C1QR	Hs.97199	Innate response	6.31	7
C6	Hs.1282	Innate response	6.22	6
GATA3	Hs.169946	Inflamation signaling	6.14	9
MMD	Hs.79889	Monocyte to macrophage	6.13	7
CCL 25	Hs.50404	Chemokine, trafficking	6.10	6
CXCL 12	Hs.237356	Chemokine, trafficking	5.86	6
CCL 27	Hs.225948	Chemokine, trafficking	5.34	6
C4BPB	Hs.99886	Innate response	5.12	6
CCR 4	Hs.184926	Chemokine, trafficking	5.11	3
CCL 4	Hs.75703	Chemokine, trafficking	4.62	3
IL 17C	Hs.110040	Chemokine, trafficking	4.25	14
IL 1β	Hs.126256	Cytokine, proinflammatory	4.20	3
MRC 2	Hs.7835	Macrophage activation	3.20	7
CXCL 9	Hs.77367	Chemokine, trafficking	3.00	2
CCL 20	Hs.75498	Chemokine, trafficking	2.88	3
CXCL 3	Hs.89690	Chemokine, trafficking	2.79	13
CXCL 2	Hs.75765	Chemokine, trafficking	2.78	3
CD 14	Hs.75627	Macrophage activation	2.73	3
IL 8	Hs.624	Chemokine, trafficking	2.70	3
NFκβ1	Hs.83428	Macrophage activation	2.61	13
CCL 22	Hs.97203	Chemokine, trafficking	2.60	3
CCR 7	Hs.1652	Chemokine, trafficking	2.55	9
***Blood Coagulation Genes:***				
SERPINA3	Hs.234726	Inflammation activation	9.39	3
SERPINI2	Hs.158308	Proteinase inhibitor	8.82	11
TBXAS 1	Hs.2001	Blood coagulation	7.70	8
FGB	Hs.7645	Fibrin	6.75	4
SERPIND1	Hs.1478	Blood coagulation	6.69	14
SERPINI1	Hs.78589	Proteinase inhibitor	6.68	6
SERPINA7	Hs.76838	Proteinase inhibitor	6.60	7
GP1bα	Hs.1472	Platelet aggregation	5.68	6
PROC	Hs.2351	Blood coagulation	5.63	6
Factor 10	Hs.47913	Blood coagulation	4.66	6
Factor 2	Hs.76530	Blood coagulation	4.50	6
GP 9	Hs.1144	Platelet aggregation	4.02	6
Factor 3	Hs.62192	Blood coagulation	3.60	2
FGG	Hs.75431	Fibrin	2.60	3
SEPPINE1	Hs.82085	Anti-Fibrinolysis	1.75	9
***Signaling Genes:***				
UNC 13	Hs.155001	Apoptosis induction	11.19	4
CARD 4	Hs.19405	Apoptosis, caspase activator	10.68	14
TNFRSF17	Hs.2556	Apoptosis signaling	10.19	14
ARL 1	Hs.242894	Cell-cell signaling	8.66	2
LGALS 3	Hs.79339	Signaling, scavenger activity	7.65	6
FAT	Hs.166994	Tumour suppressor	7.15	2
TNFSF9	Hs.1524	Apoptosis signaling	6.85	6
BBC3	Hs.87246	Apoptosis signaling	6.77	9
TNFRSF13β	Hs.158341	Apoptosis signaling	6.49	6
DDX24	Hs.155986	Apoptosis signaling	6.11	6
TNFRSF11β	Hs.81791	Apoptosis signaling	6.11	7
TNFRSF10β	Hs.51233	Apoptosis signaling	6.09	6
BNIP2	Hs.155596	Apoptosis signaling	5.81	1
MGST 2	Hs.81874	Cell-cell signaling	5.50	6
CUL 4	Hs.183874	Tumour suppressor	5.35	6
BAF 53A	Hs.274350	Cell-cell signaling	5.26	6
MAPK 1	Hs.324473	Apoptosis induction	5.11	14
CUL 2	Hs.82919	Tumour suppressor	5.09	6
P2RX 1	Hs.41735	Apoptosis, ion channel activity	4.43	6
TYROBP	Hs.9963	Intracellular receptor signaling	2.51	13
CNIH	Hs.201673	Intracellular signaling	2.13	9
ADRα1A	Hs.52931	Cell-cell signaling	2.08	3
DAPK 1	Hs.153924	Apoptosis induction	2.01	9
***Defense-related Genes:***				
TFF 1	Hs.350470	Defense response, maintenance	7.65	9
TFF 3	Hs.82961	Extracellular defense response	6.98	7
GAGE 1	Hs.128231	Cellular defense response	6.82	6
DEFβ3	Hs.283082	Extracellular defense response	6.01	5
RU 2	Hs.61345	Extracellular defense response	5.83	6
DEFα4	Hs.2582	Extracellular defense response	5.81	6
***Interferons-related Genes:***				
IFNαR1	Hs.1513	JAK-STAT cascade, recptor	5.41	8
IRF 7	Hs.166120	Transcription regulation	5.05	6
IF 144	Hs.82316	Invasive growth response	2.69	5
IRF 6	Hs.11801	Transcription regulation	2.06	14
IL 18	Hs.83077	Angiogenesis	1.76	6
ISGF3γ	Hs.1706	Regulatory	1.28	9
IRF 5	Hs.334450	Transcription regulation	1.23	9
IFNαR2	Hs.86958	Receptor activity	1.06	2
IFNγR1	Hs.180866	Receptor activity	0.80	5
IFNγ	Hs.856	Growth regulation	0.53	4
IRF 3	Hs.75254	Transcription regulation	0.40	5
IRF 2	Hs.83795	Transcription regulation	0.32	4
IRF 1	Hs.80645	Transcription regulation	0.30	3

Order is arranged from the highest to lowest value based on the expression range (log_2_).
