# Supplementary figures and images for: A human in vitro model system for investigating genome-wide host responses to SARS coronavirus infection
Source: BMC Infect Dis. 2004 Sep 9;4:34. doi: 10.1186/1471-2334-4-34 (PMC518965; doi:10.1186/1471-2334-4-34)

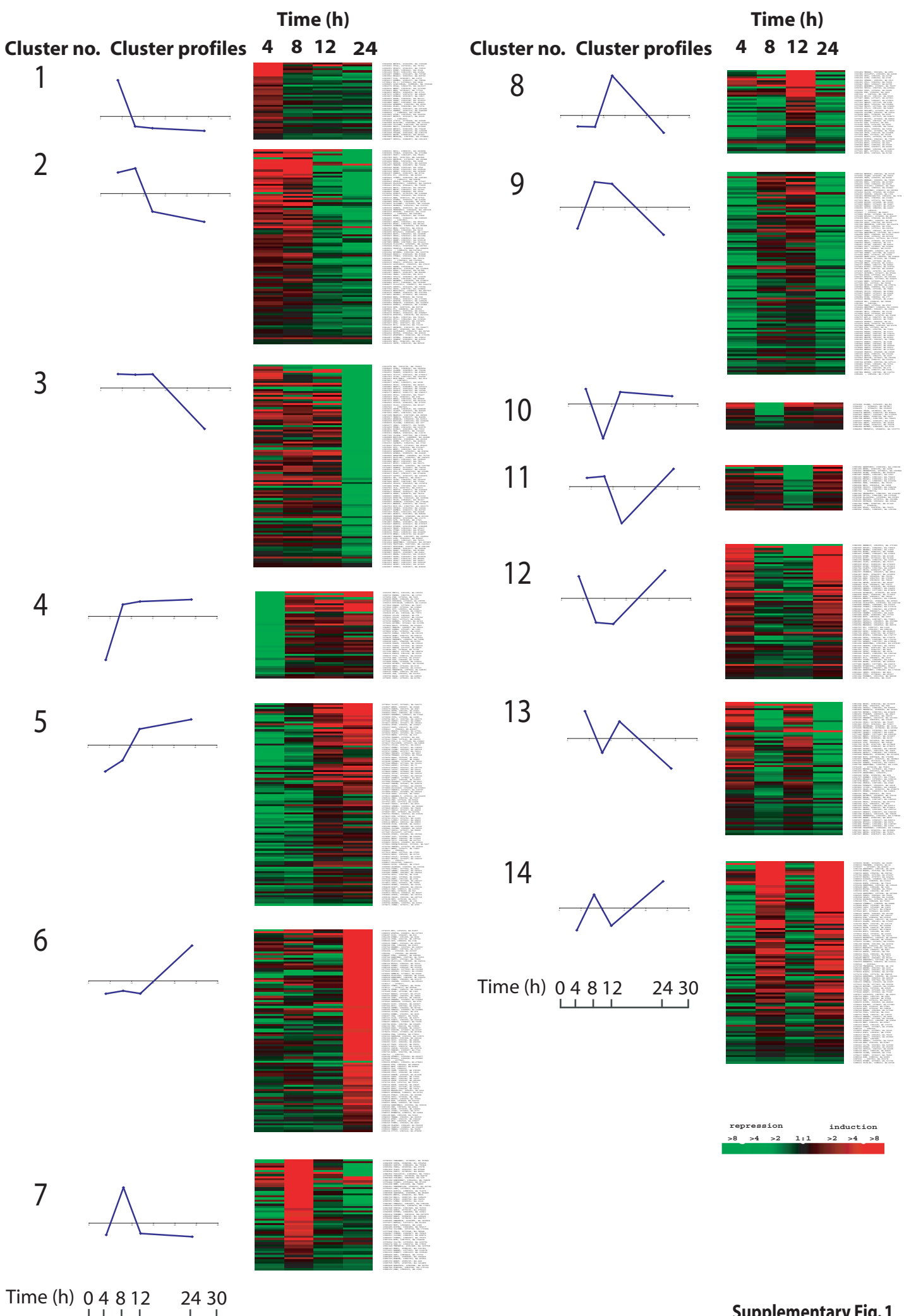

Supplement: Additional File 1 — List of all immune related genes after SARS-CoV infection Comprehensive list of 1087 immune related genes that were altered in PBMCs in response to SARS-CoV infection at 4 hours, 8 hours, 12 hours, and 24 hours. Genes were grouped and ordered using the algorithm described in Methods. Rows represent individual genes, columns represent individual time points. Each cell in the matrix represents the mean expression level from 3 subjects for a gene feature at a particular time point (non-infected PBMCs responses have been subtracted from infected responses). The red and green color bars reflect high and low expression levels respectively, while black indicates equivalent expression level. The magnitude of the log-transformed ratio is reflected by the degree of color saturation. The line graph indicates the average expression ratios for each group. The area above the axis indicates upregulation, while the area under the axis means downregulation. [file 1471-2334-4-34-S1.pdf]
